# Polyelectrolyte Multilayers on Soft Colloidal Nanosurfaces: A New Life for the Layer-By-Layer Method

**DOI:** 10.3390/polym13081221

**Published:** 2021-04-09

**Authors:** Ana Mateos-Maroto, Irene Abelenda-Núñez, Francisco Ortega, Ramón G. Rubio, Eduardo Guzmán

**Affiliations:** 1 Departamento de Química Física, Facultad de Químicas, Universidad Complutense de Madrid, Ciudad Universitaria, s/n-28040 Madrid, Spain; ana.mateos@ucm.es (A.M.-M.); irenabel@ucm.es (I.A.-N.); fortega@ucm.es (F.O.); rgrubio@quim.ucm.es (R.G.R.); 2 Instituto Pluridisciplinar, Universidad Complutense de Madrid, Paseo Juan XXIII, 1-28040 Madrid, Spain

**Keywords:** layer by layer, polyelectrolytes, electrostatic self-assembly, multilayers, soft colloids, nanosurfaces

## Abstract

The Layer-by-Layer (LbL) method is a well-established method for the assembly of nanomaterials with controlled structure and functionality through the alternate deposition onto a template of two mutual interacting molecules, e.g., polyelectrolytes bearing opposite charge. The current development of this methodology has allowed the fabrication of a broad range of systems by assembling different types of molecules onto substrates with different chemical nature, size, or shape, resulting in numerous applications for LbL systems. In particular, the use of soft colloidal nanosurfaces, including nanogels, vesicles, liposomes, micelles, and emulsion droplets as a template for the assembly of LbL materials has undergone a significant growth in recent years due to their potential impact on the design of platforms for the encapsulation and controlled release of active molecules. This review proposes an analysis of some of the current trends on the fabrication of LbL materials using soft colloidal nanosurfaces, including liposomes, emulsion droplets, or even cells, as templates. Furthermore, some fundamental aspects related to deposition methodologies commonly used for fabricating LbL materials on colloidal templates together with the most fundamental physicochemical aspects involved in the assembly of LbL materials will also be discussed.

## 1. Introduction

The fabrication of nanomaterials with controlled structure and specific functionalities is currently a challenge for several industrial and technological fields [1,2,3,4,5,6]. This requires tools enabling a precise control of the self-assembly and self-organization of molecules and colloidal objects at the molecular level (nanoscale), and the possibility to modulate such processes by external fields/stimuli [7,8,9,10]. The Layer-by-Layer (LbL) assembly technology offers many possibilities for fulfilling the above-mentioned requirements by the combination of a broad range of functional building blocks into macromolecular devices [11,12]. The combination of this versatility with their simplicity, modularity, and low cost allows manufacturing multilayered materials with controlled thickness and composition, and tunable properties and structure. This has fostered the development of the LbL technology as one of the most extended approaches for the fabrication of nanostructured functional materials (films, membranes, and capsules) in the last decades [13,14,15,16]. The importance of the LbL method is reflected in the high number of publications related to this fabrication methodology (more than 1000 per year) and its use in different commercial products, e.g., contact lens (Ciba Vision, Duluth, GE, USA) or coatings for chromatography column (Agilent Technologies, Santa Clara, CA, USA) [17].

The simplest LbL approach is based on the fabrication of ordered films by the sequential deposition of two mutual interacting species on a substrate, which works as a template for the assembly process. The seminal works on the assembly of LbL films were focused on the use of conventional flat macroscopic surfaces as a substrate (mainly glass slides, quartz plates, or silicon wafers) [18]. However, the development of the LbL approach allows expanding the type of the substrates beyond traditional macroscopic flat surfaces, and currently, the fabrication of LbL materials on colloidal micro/nanoparticles (hard and soft particles), liposomes or vesicles, micelles, fluid interfaces (floating multilayers), emulsion droplets, or even cells is possible [19,20,21,22,23,24,25,26,27,28]. Furthermore, the richness of the chemical composition and physical properties (e.g., size, geometry, shape, or roughness) of the substrates make that the LbL can be considered one of the most prominent tools of the interfacial nano-engineering for manufacturing advanced functional materials [29]. Even though the LbL approach was initially introduced for the fabrication of the so-called polyelectrolyte multilayers (PEMs) through the electrostatically-driven deposition of alternate layers of oppositely charged polyelectrolytes, i.e., polyanions and polycations, or bolaamphiphiles [18,30], nowadays, it is possible to assemble LbL materials through different types of interactions, e.g., hydrogen bonding [31,32], charge transfer interactions [33], molecular recognition [34,35], coordination interactions [36], chiral recognition [37], host–guest interactions [38], π–π interactions [39], biospecific interactions [40], sol–gel reactions [41], or even covalent bond (“click chemistry” reactions) [42,43], with the mutual interaction between the alternately assembled blocks being the only condition for the assembly of LbL materials [6]. This has increased the number and nature of the materials used in the LbL films, i.e., the building blocks, and currently, the list includes a broad range of charged and uncharged molecules and colloidal nano-objects (organic and inorganic), e.g., synthetic oppositely charged polyelectrolytes (both strong and weak polyelectrolytes); synthetic neutral polymers poly(ethyleneglycol) or poly(vinylpirrolidone); colloidal particles and nano-objects (graphene and graphene oxide nanoplatelets, carbon nanotubes, dendrimers, clays, microgels, polymeric, ceramic, or metallic particles); biomolecules (proteins and peptides, polysaccharides, nucleic acid, lipids); dyes; viruses; and even, in some cases, molecular species [44,45,46,47,48,49,50,51,52,53,54,55,56,57,58,59,60,61,62,63,64,65,66,67,68,69]. Therefore, the LbL assembly technology can be easily modulated for their adaption to the specific nature of a broad range of building blocks and substrates [70,71].

The innovation in the materials and assembly technologies, together with the increase of the techniques for film characterization have fueled the intense and growing interest on the fabrication of LbL materials [11,29]. Nowadays, the LbL approach allows one to assemble different types of devices/materials with controlled size, shape, and morphology, ranging from flat films and nano/microcapsules [14,25,72,73] to a broad range of sophisticated systems, including multicapsules with several hierarchically organized nanocontainers, onion-like structures, sponges, membranes, or nanotubes [74,75,76,77,78,79,80,81,82,83]. This broad range of supramolecular architectures combined with the possibility to introduce stimuli responsiveness on the obtained materials, i.e., the assembled systems can shrink, swell, burst, or reconfigure upon the application of a certain physicochemical stimuli (e.g., pH, heat, solvents, mechanical stresses, or salts) has driven the advance on the understanding and application of the LbL method [84,85,86,87,88,89,90].

The last decades have been really fruitful in research efforts aimed to shed light on the foundations and fringes of the LbL method, as well as on the development of new applications of the built materials [11,12,14,18,29,30,91,92,93,94,95,96]. This review is aimed to spotlight some of the most relevant aspects of the fabrication of LbL multilayers on liposomes, emulsion droplets, or cells (soft colloidal nanosurfaces). The use of soft colloidal nanosurfaces as a template for the assembly of LbL materials has gained importance in the last years mainly because the assembled materials can be easily used for obtaining capsules with tailored properties and containing different functionalities [79]. Therefore, the LbL approach may allow a rational design and fabrication of a new class of engineered materials, which can find applications in several research fields, ranging from medicine and biotechnology to catalysis or synthetic chemistry.

## 2. A Brief Analysis of the Physicochemical Foundations of Polyelectrolyte LbL Assembly

The use of the LbL method for the assembly of multilayered films requires analyzing some of the physicochemical aspects underlying the control of the properties and structure of the films [11,97]. Among such aspects, the understanding of the driving forces guiding the alternate layer deposition and the multilayer growth may be accounted as to important aspects for controlling the assembly of devices using the LbL approach. It should be noted that the understanding of such aspects requires more knowledge of how the bath conditions (ionic strength, chemical nature of the supporting electrolyte, temperature, and pH), assembly procedure (drying and rinsing steps, and deposition time for the layers), and the nature of the building blocks impact the assembly process [97].

### 2.1. Understanding the Polyelectrolyte Multilayer Growth 

The assembly of LbL films using polyelectrolytes takes advantages of the ability of polyelectrolytes for self-organizing in supramolecular structures together with their capacity to form complexes when oppositely charged polyelectrolytes are mixed [18,30,91,98,99,100]. This is an advantage for obtaining LbL materials where the thickness is tuned almost at will. The possibility to control the thickness is essential for optimizing different applied aspects of the LbL materials, e.g., the transparency of optical devices, retention of encapsulated drugs, or wetting properties of the surfaces and adhesion.

**Figure 1:**
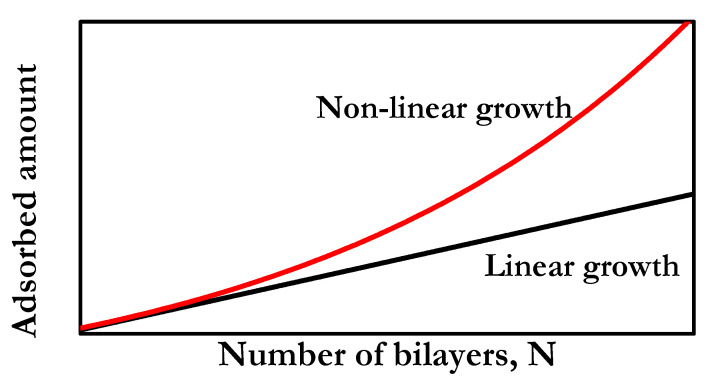
Sketch showing an idealization of the linear and non-linear trends of polyelectrolyte multilayers as the dependence of the adsorbed amount on the number of bilayers, N.

It is generally assumed that there is the possible existence of two different types of trends for the change of the adsorbed amount (or layer thickness) on the number of bilayers, N, i.e., the growth mechanism in LbL materials: linear and non-linear (*Note: the non-linear growth is generally called in the literature exponential growth. However, the dependence of the adsorbed amount on the number of bilayer is not always truly exponential*). In the following, multilayers will be denoted as (A–B)_n_, with A and B representing the interacting species, and the subindex *n* representing the number of bilayers. Figure 1 displays a schematic picture of the two common growth dependences found in LbL films.

Linear growth appears when the adsorbed amount follows a quasi-linear dependence with the number of bilayers; i.e., the adsorbed amount per bilayer remains constant. This results in the formation of multilayers in which the thickness increases only a few nanometers upon bilayer deposition, with this thickness increase being about the sum of the characteristic lengths of polycation and polyanion. It is worth mentioning that the thickness and, in many cases, the growth dependence can be tuned almost at will by the modification of the assembly conditions, i.e., the interaction balance involved in the assembly process. Paradigms of multilayers following a linear growth are (PAH-PSS)_n_ one (with PAH and PSS being poly(allylamine hydrochloride) and poly(4-styrenesulfonate of sodium), respectively) and those built by the deposition of PDADMAC and PSS from solution of low ionic strength (with PDADMAC being poly(diallyldimethylammonium chloride). Other linear growth multilayers are (PAH-PAA)_n_ and (PM2VP-PSS)_n_ (with PAA and PM2VP being poly(acrylic acid) and poly(N-methyl-2-vinyl pyridinium chloride), respectively) [101]. On the other side, non-linear growth is associated with an increase faster than linear, i.e., supra-linear dependence, with the deposition of each bilayer. Therefore, the variation of the thickness is not the same per each bilayer, and consequently, the multilayer growth does not depend on the characteristic lengths of the assembled molecules themselves. (PDADMAC-PSS)_n_ multilayers assembled from solutions in which the effective charge of the polyelectrolyte is low (generally solutions with high ionic strength) may be considered as a paradigm of non-linear growth [89,102,103,104]. Furthermore, there are many other examples of multilayer following a non-linear growth, with this multilayer being formed in most of the cases for biopolyelectrolytes, e.g., (CHI-PAA)_n_, (PLL-HA)_n_, or (PLL-PGA)_n_ (with CHI, PLL, HA, and PGA being chitosan, poly(L-lysine), hyaluronic acid and poly(glutamic acid), respectively) [46,47,105,106,107]. It should be noted that linear and non-linear growth are not the only growth tendencies that appear in polyelectrolyte LbL multilayers, and it can be possible to find, for specific combinations of polyelectrolyte pairs, exotic growth dependences in which the typical rules driving the assembly of LbL polyelectrolytes are violated [108,109].

It should be noted that even though the assembly of polyelectrolyte multilayers using the LbL approach appears to be a well-established field accounting for three decades of research effort, there is currently a strong controversy about the physical mechanisms governing the emergence of different types of dependences of the adsorbed amount on the number of bilayers. In the following, a critical discussion on the most fundamental aspects of the current knowledge on the polyelectrolyte multilayer growth is included. The main differences on the growth mechanisms are commonly associated with the existence of diffusion of the adsorbing molecules within the multilayer structure.

The seminal work trying to provide a physical reliable accounting for the differences on the multilayer growth was performed by Elbert et al. [110]. They reported that polyelectrolytes can diffuse within the multilayer structure either in linear growth multilayers or in a non-linear one. However, the diffusion process appears slightly different depending on the type of multilayer growth. For linear growth multilayers, the adsorption process involves an initial deposition of the molecular polyelectrolyte layers followed by the diffusion of some chains to the inner region of the multilayer, which leads to a strong intermingling of the layers, resulting in the formation of polyelectrolyte blend layers. This agrees with the theoretical description provided by Subbotin and Semenov [111] using a mean field approach.

The ability for diffusion of the polyelectrolyte together with the final thickness of the multilayer are closely correlated to the polyelectrolyte molecular weights and charge densities. On the other side, Elbert et al. [110] suggested that the assembly of non-linear growth multilayers is associated with the adsorption of more than one layer in each deposition cycle. This is counterintuitive due to the strong repulsion between polyelectrolyte chains. However, the existence of the diffusion of the polyelectrolyte chains within the multilayer structure facilitates the deposition of more than one layer per deposition cycle. This is possible for specific polyelectrolyte pairs and assembly conditions through a coacervation-like process between polyelectrolyte in solution and the polyelectrolyte bearing opposite charge diffusing from the inner region multilayer to the solution–multilayer interface. The coacervates formed at the interfacial region may precipitate justifying an increase of the thickness above that expected for a polymer molecular layer [112,113]. Thus, the multilayer behaves as a polymer reservoir, and the increase of the thickness as the multilayer growth increases the number of polymer chains diffusing to the solution–multilayer interface, and as a matter of fact, it increases the coacervate formation (*Note that the concentration of the solution used for layering is commonly several times that required for forming a molecular layer*). The above picture agrees with the theoretical studied by Tang and Besseling [114], in which they proposed a correlation between the formation processes of inter-polyelectrolyte complexes in solution and polyelectrolyte multilayers. Thus, it may be expected that the differences on the growth mechanisms are related to differences on the dynamics of the polyelectrolyte chains and in particular to changes on the ionic pairing on the multilayers [115].

The description of the origin of the non-linear growth proposed by Elbert et al. [110] agrees with the works by Picart et al. [105,106,116,117,118]. They associated the emergence of non-linear growth with an *in* and *out* diffusion of at least one of the polyelectrolyte within the multilayer structure, neglecting the role of any diffusion on linear growth films. The latter can be ascribed to the fact that no control experiments including linear growth multilayers were performed [105,117,119]. In particular for the case of (PLL–HA)_n_ multilayers, diffusion of the PLL chains within the multilayer structure was found, with this diffusion being controlled by the nature of the layering solution. Thus, the exposure of multilayers to PLL solutions results in its diffusion to the inner region of the multilayer, with the opposite phenomena being found upon exposure of the multilayers to HA solutions. The latter results in a process of PLL-HA complexation at the solution–multilayer interface, which results in the adsorption of a higher amount of material than that expected for a single monolayer, and consequently, the bilayer appears thicker than that expected, considering the adsorption of true molecular layers. Further details on the role of the *in* and *out* diffusion on the emergence of non-linear growth were obtained by confocal laser scanning microscopy (CLSM) [117]. Thus, it is possible to monitor the adsorption process within the axis perpendicular to the multilayer by using fluorescently labeled polyelectrolytes at different heights of the multilayer. CLSM experiments pointed out the existence of diffusion of fluorescently labeled PLL within the entire multilayer, whereas the fluorescently labeled HA chains remained trapped at the same positions where they were initially assembled. These results confirmed the existence of interdiffusion of at least one of the polyelectrolyte in non-linear growth multilayers, or both as was found for (PLL-PGA)_n_ films [120]. However, they do not provide any favorable or unfavorable evidences in relation to the existence of similar processes on linear growth multilayers. The existence of *in* and *out* diffusion of the polyelectrolyte chains may be explained in terms of the existence of Donnan potential within the LbL film. This potential emerges from the charge excess originated from the mobile chains, and hence the interdiffussion occurs until the equilibration of such potential [116,121]. The above discussion indicates the importance of the interdiffusion processes on non-linear growth multilayers. However, it does not provide an appropriate explanation of their role in linear growth multilayers. Furthermore, it appears as inconsistent for accounting the assembly of polyelectrolyte multilayers where a transition from linear to non-linear growth occurs as result of a modification of the assembly conditions, e.g., (PDADMAC-PSS)_n_, [89,102,122]. This may be accounted for the picture proposed by Yuan et al. [123]. They proposed that the emergence of non-linear growth may be associated with a loose ionic cross-linking, and the existence of a preferential interaction of the polycation with the polyanion deposited in the film may favor the interdiffusion of the polycation chains, and consequently the emergence of non-linear growth. This agrees with the enhanced interdiffusion found by Guzmán et al. [124] associated with the ionic strength driven transition from linear to non-linear growth in (PDADMAC-PSS)_n_, and it does not limit the interdiffusion to non-linear growth. Furthermore, Yuan et al. [123] reported the importance of the specific chemical nature of the polymer in the control of the interdiffusion process.

Guzmán et al. [124], by combining structural characterization of the multilayers and a rigorous analysis of the adsorption kinetics of the polyelectrolyte layers, demonstrates that the interdiffusion is not the characteristic difference between linear and non-linear growth. They proposed that the increase of the film roughness upon layer deposition may be an essential driving force for the emergence of non-linear growth, which may be correlated to the deposition of coacervates proposed by Elbert et al. [110]. Thus, the increase of the roughness may be associated with an increase of the area available for the adsorption, and consequently with an increase of the amount of polyelectrolyte deposited in each bilayer, which as matter allows a multilayer growth faster than linear. On the other side, the deposition of chains respecting its molecular size in each deposition cycle results in a linear growth [102,122,124,125,126]. The effect of the roughness as an essential parameter for controlling the type of multilayer growth is compatible with the model proposed by Haynie et al. [127], in which the non-linear growth is considered the result of a propagation, growth, and coalescence of dendritic or isolated aggregates. This leads to an increase of the film roughness with each adsorption step and consequently to the non-linear growth of the multilayer. Hernández-Montelongo et al. [128] verified experimentally, using fractal analysis on Atomic Force Microscopy images, the role of the dynamics of layer adsorption and the formation of heterogeneous structures on the control of the multilayer growth. They found that linear growth multilayers assemble by the aggregation of the deposited molecules followed by a rearrangement process, whereas the non-linear growth is characterized by a diffusion limited aggregation. This leads to the formation of isolated aggregates as result of diffusion gradients, which results in a significant increase of the multilayer roughness. On the other side, multilayers exhibiting linear growth appear smooth and with regular surfaces. The emergence of a heterogeneous growth, at least during the initial stages of the multilayer assembly, was also reported by Picart et al. [105] in the assembly of (PLL-HA)_n_ multilayers. However, they neglect the implication of the emergence of heterogeneous structures in the emergence of non-linear growth on polyelectrolyte multilayers. Very recently, Naas et al. [129] has shown using electron paramagnetic resonance spectroscopy that the appearance of different types of growth in polyelectrolyte multilayers results from a combination of different aggregation patterns of polyelectrolyte multilayers upon the deposition and modifications on the adsorption dynamics.

The importance of the adsorption time on the emergence of non-linear growth in polyelectrolyte multilayers was furtherly extended by Selin et al. [130] and Sustr et al. [119]. They suggested that the growth rate (linear or non-linear) depends strongly on the specific diffusivities of the assemble block, with the polyelectrolyte uptake and consequently the type of growth being strongly correlated to the contact time and the film thickness.

The above picture makes it clear that a true understanding of the origin of the differences on the growth of polyelectrolyte multilayers is far from clear. However, the current knowledge does not allow considering the interdiffusion as a distinctive signature defining the growth type. Therefore, the understanding of the type of growth appearing on a specific multilayer can be only obtained examining carefully the specific pair of assembled polyelectrolytes, their conformations, and the conditions used for the assembly of the multilayers.

### 2.2. Charge Compensation and Charge Inversion 

The direct electrostatic interactions between polyelectrolytes bearing opposite charges deposited in adjacent layers should be expected to present a very central role on the control of the formation of LbL multilayers. However, the true picture involves a more complex situation where different types of interactions are involved: polyelectrolyte–polyelectrolyte, polyelectrolyte–solvent, and polyelectrolyte–template [103,131]. The understanding of the complex interplay of interactions makes necessary a careful examination of two main aspects that enable modulating the assembly process: (i) quality of the solvent for the polyelectrolytes (ionic strength, pH, or temperature), and (ii) competence between electrostatic and entropic factor [97,102,122].

The deposition of polyelectrolytes, or other charged molecules, onto oppositely charged surfaces is commonly associated with the emergence of a charge inversion phenomena, i.e., the adsorption is not stopped once the neutralization of the surface charge is reached, proceeding until a certain degree of charge with the same sign as that of the layering species appears on the surface [132]. This may be understood considering the role of the steric hindrance, which makes the neutralization of the initial charge of the surface difficult, and hence the adsorption of some additional charged molecules are required to ensure a true neutralization of the charged surface. Thus, the adsorption of a polyelectrolyte onto a surface bearing an opposite charge results in a charge inversion phenomena and the formation of a fuzzy layer with charged segments (loops and tails) protruding to the solution. This leads to an overcompensation of the initial charge of the surface, which hinders the further adsorption of molecules due to the repulsion appearing between the multilayer and the solution molecules with the same sign, i.e., polyelectrolyte adsorption onto the charge surfaces appears as an electrostatically self-limited process. The incorporation of additional layers on the multilayer will follow a similar pathway to the above described, with the overcompensation of the charge of the previously deposited layer playing a central role on the alternate deposition of the subsequent layers [21,47,102,133]. Despite the apparent counterintuitiveness of the charge inversion phenomenon, it plays a very central role either for the electrostatic self-assembly of LbL films [103] or for many other processes with interest for materials science and biology [134].

**Figure 2:**
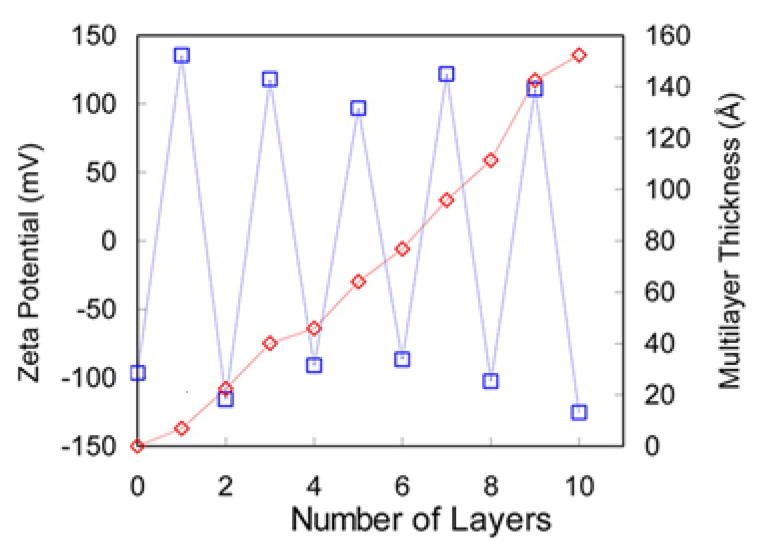
Change of the ξ potential (blue symbols) and multilayer thickness (red symbols) with the alternate deposition of poly(diallyldimethylammonium chloride (PDADMAC) and poly(4-styrenesulfonate of sodium) (PSS) layers onto silicon wafers from polyelectrolyte solutions with concentration 10 mM, and ionic strength fixed in 100 mM. Reprinted from reference [139], Copyright (2014), with permission from the American Chemical Society.

Evidence of the overcompensation on LbL films has been found by measuring different physicochemical parameters related to the effective charge of the surface, e.g., surface potential, streaming potential, or zeta potential [21,47,89,102,103,105,135,136,137,138]. These techniques provide information on the oscillation of the surface charge between positive and negative values with the alternate stacking of polycation and polyanion layers. Figure 2 shows the change of the zeta potential for (PDADMAC-PSS)_n_ deposited onto silicon wafers [139], with the overcompensation being evidenced from the oscillation of the zeta potential values between values about +100 and −100 mV upon the deposition of polycation and polyanion layers, respectively. It is worth mentioning that even though Figure 2 presents evidence of a symmetric overcompensation in polyelectrolyte layers bearing opposite charges, in most of the cases, the asymmetrical growth of the films results in an overcompensation dependent on the nature of the deposited layer [140].

The charge inversion appears as a self-limiting process depending exclusively on the specific nature of the pair of assembled polyelectrolytes, and only a minimal effect of the assembly conditions (e.g., ionic strength or pH) on the maximum overcompensation degree can be expected [102,122]. The overcompensation is at a maximum at the surface layer, and there is decay toward the inner region of the multilayers [89,103]. However, the extension of the overcompensation is correlated to the multilayer growth and fuzziness, with non-linear multilayers exhibiting overcompensation within the whole deposited layer [106], whereas overcompensation is limited to the layer surface for linear growth multilayers [13]. 

The overcompensation can be explained considering that the deposition process does not lead to a perfect matching between the charges of the monomers in adjacent layers, appearing a charge excess that must be compensated for avoiding the instability of the film and fulfilling the electroneutrality boundary conditions [89,141]. Therefore, a contribution counter-balancing the charge excess is mandatory to result in multilayers with a zero net charge at the macroscopic scale (beyond the Debye length). This makes it necessary to incorporate small ions in the multilayer to compensate the excess of charge appearing from the unpaired polyelectrolyte segments of the layers [102,104,142,143], which results in two different compensation mechanisms depending on the contribution of the small ions in ensuring the electro-neutrality of the multilayer [144,145,146,147]: (i) intrinsic, and (ii) extrinsic [141].

**Figure 3:**
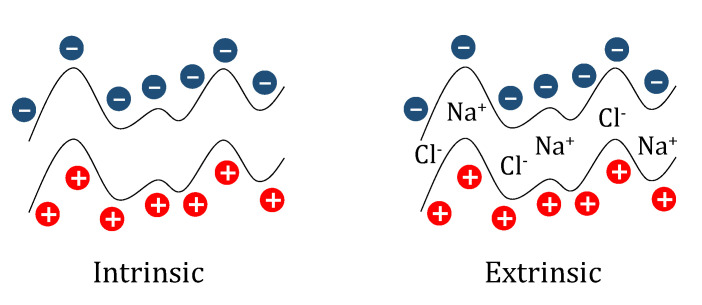
Scheme of the expected configuration of polyelectrolyte layers and counterions in intrinsically and extrinsically compensated polyelectrolyte multilayers.

The intrinsic compensation is associated with a perfect matching between the polyelectrolyte charges in adjacent layers, and hence to ensure the ionic equilibrium within the multilayer the assembly of the polyelectrolyte layers expels any small ions from the vicinity of the film. This results in the formation of multilayers with stoichiometry 1:1 (polycation:polyanion), which presents a significant degree of ionic cross-linking between polymer chains. Furthermore, the expulsions of counterions from the vicinity of the multilayers is associated with a favorable entropic contribution to the decrease of the average free energy of the systems, with the entropy gain resulting from the counterion release process becoming the main driving force for the assembly process. The scenario is significantly different when there is not a perfect matching between the charges in adjacent layers, with the presence of counterions being mandatory for ensuring the electroneutrality of the multilayers. This results in the so-called extrinsic compensation, which leads to the formation of multilayered structures with a broad range of different stoichiometries and to an almost negligible role of the entropy on the assembly of the multilayer. Figure 3 shows a sketch representing an idealized picture of the distribution of polyelectrolytes and counterions depending on the type of compensation involved in the multilayer stabilization. It should be stressed that purely intrinsic compensation scarcely appears in polyelectrolyte multilayers (only in polyelectrolytes with a very high charge density) [89,140,148], and most of the multilayers present extrinsic-like compensation [45,46,47,89,103,104]. However, there are no rules enabling the prediction of the type of compensation expected for the assembly of a specific pair of polyelectrolytes and a specific set of assembly conditions [22,45,46,47,89,103,104].

The compensation mechanism can be tuned by modifying any parameter impacting on the ionic equilibrium within the multilayer, which influences both the final structure and the physicochemical properties of the LbL multilayered films [89,102,103]. Among the parameters contributing to the ionic equilibrium, the ionic strength is possibly that with the strongest impact on the modulation of the compensation mechanism due to its ability for modulating the effective charge density of the polyelectrolyte by ionic condensation [140]. This was verified from the studies by Schlenoff and Dubas [103] on (PDADMAC-PSS)_n_ multilayers, where they demonstrated that the compensation mechanism of the multilayers may be switched from a mainly intrinsic-like compensation to a true extrinsic one. Thus, at low ionic strength, the layer assembly proceeds with a noticeable release of counterions from the vicinity of the film to the solution, which is associated with a very favorable entropic contribution to the assembly process, whereas at high ionic strength, the high amount of ions on the bulk leads to a situation where the contribution of the counterion release from the multilayer plays a very limited role on the entropy gain, and hence a high proportion of counterions remain embedded on the multilayers, resulting in an extrinsic-like compensation. Therefore, it is possible to assume that the energetic landscape is very different for intrinsic and extrinsic compensated films due to the different role of the entropy gain associated with the counterion release [89,102]. The type of compensation can be evaluated in terms of the compensation ratio, *R_c_*, that provides a measurement of the ratio between the density of positively, $\rho_{monomer}^{+}$, and negatively, $\rho_{monomer}^{-}$, charged monomers in adjacent layers [45]

(1) $$R_{c}=\frac{\rho_{monomer}^{+}}{\rho_{monomer}^{-}}$$

**Figure 4:**
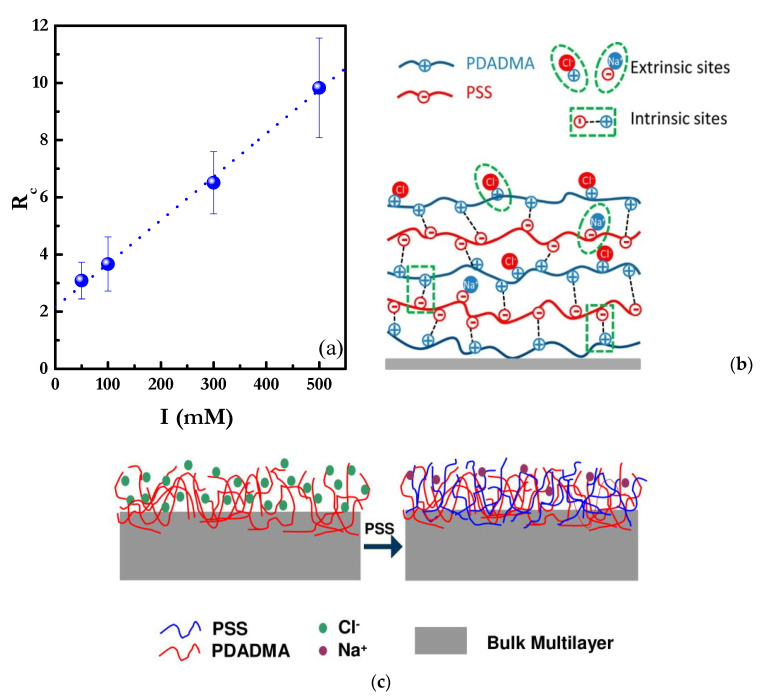
(**a**) Compensation ratio *R_c_* in (PDADMAC-PSS)_n_ multilayers as a function of the ionic strengths. Data adapted from reference [89], Copyright (2009), with permission from The Royal Society of Chemistry. (**b**) Sketch displaying the general internal charge balance in polyelectrolyte multilayers. Reprinted from reference [140]. Copyright (2013), with permission from American Chemical Society. (**c**) Representation of the asymmetrical compensation in polyelectrolyte multilayers as a function of the nature of the last deposited layer. Reprinted with permission from reference [149]. Copyright (2012) American Chemical Society.

According to Equation (1), values of *R_c_* close to 1 indicate a mostly intrinsic compensation, whereas values of *R_c_* above or below 1 evidence the extrinsic compensation of the films with an excess of cationic or anionic monomers, respectively. Figure 4a shows the effect of the ionic strength on the type of compensation mechanism for (PDADMAC-PSS)n multilayers in terms of the dependence of *R_c_* on the ionic strength (*I*) [102]. The values of *R_c_* indicate the existence of an excess of PDADMAC monomers in relation to PSS one in adjacent layer, which is a clear signature of extrinsic-like compensation. Furthermore, the increase of *R_c_* with the ionic strength evidences the transition from a quasi-intrinsic compensation to a clearly extrinsic compensation. Furthermore, the values of *R_c_* give an indication of the asymmetric character of the layer compensation, i.e., the nature of the capping layer defines the degree of extrinsic compensation within the multilayer [89,149]. This is clear considering that *R_c_* values above one are related to the existence of a higher charge excess in the PDADMAC-capped layer than in those capped with PSS, and hence, it may be expected for there to be a clearly extrinsic compensation for PDADMAC layers, whereas the compensation becomes mainly intrinsic when PSS layers are considered. This asymmetric compensation depends on the specific nature of the polyelectrolyte pair, and it can influence decisively on the layer structure and physicochemical properties. Thus, in the particular case of (PDADMAC-PSS)_n_ multilayers, the counterion distribution profile affects the osmotic stress within the multilayer, which results in the formation of swelled and highly hydrated PDADMAC layers and a more collapsed PSS layer. This affects the roughness of the layers, with the PDADMAC-capped films having higher roughness than the PSS-capped one [89,150] (see inset, Figure 4a). Therefore, the internal charge balance in polyelectrolyte multilayers involves a combination of both extrinsic sites (polyelectrolyte/counterion pairing) and intrinsic sites (pairing between oppositely charged polyelectrolytes), as is schematized in Figure 4c.

It should be noted that the differences of the compensation mechanism associated with the specific nature of the polyelectrolyte pair and the assembly conditions are related to differences on the forces driving the assembly, i.e., the role of enthalpic and entropic contributions to the assembly process [151]. For the assembly of (PDADMAC-PSS)_n_ multilayers, a strongly exothermic complexation is found for multilayers assembled from solutions having low ionic strength, whereas the complexation becomes endothermic as the ionic strength increases [11]. Thus, considering a change of Gibbs energy for the assembly process given Δ*G* = Δ*H* − *T*Δ*S*, *it may be expected that both enthalpy* Δ*H* (negative as result of the ionic pairing) and entropy Δ*S* (positive as result of the counterion release) presents a favorable impact for the assembly at low ionic strength. On the other side, enthalpy and entropy present a counteractive impact for the assembly, which can result in deconstruction phenomena of the multilayers as the ionic strength is increased [127,152].

The entropic balance of the assembly is not only associated with the ionic equilibrium, as two other main contributions exist that may influence such balance: (i) entropy associated with the release and reorientation of hydration water [153,154,155], and (ii) entropy penalty associated with the reduction of the degrees of freedom of the molecules due to their attachment to the surface [156,157]. However, the impact of these two contributions, in most of the systems, is smaller than that corresponding to the counterion release, which allows one to neglect their role as a driving force of the assembly [158].

The importance of the entropy as a driving force of the LbL assembly of polyelectrolyte multilayers can be better understood considering three main points: (i) adsorption beyond the zero net surface charge point is not possible without the contribution of non-electrostatic interactions because of the high enthalpic penalty associated with charge inversion; (ii) electrostatic interactions do not differ between intrinsic (compensation by ionic pairing) and extrinsic compensations (compensation by counterions condensation), and (iii) multilayers can be assembled using materials with reduced charge density or even under conditions where the electrostatic interaction is more or less screened, e.g., high ionic strength [47,89,141,159,160,161,162,163].

## 3. Fabrication of LbL Assemblies on Colloidal Surfaces

### 3.1. Hard and Soft Colloids as LbL Templates

The colloidal template used for the LbL determines in most of the cases the shape, morphology, and properties of the multilayered films, and it can be classified into two main groups: hard and soft [164]. Among the former, melamine formaldehyde, polystyrene, SiO_2_, and CaCO_3_ particles are probably the most commonly used [165,166]. The use of SiO_2_ particles as a template is particularly interesting because it allows fabricating extremely monodisperse capsules. However, their true application is very limited, because the dissolution of the SiO_2_ core requires hydrofluoric acid for obtaining hollow capsules. The use of polystyrene and melamine formaldehyde particles as a template remain an important challenge because their complete dissolution is difficult, and some residues remain upon chemical attachment, which makes their application on encapsulation for biomedical purposes almost impossible. The use of CaCO_3_ particles deserves particular attention because their biocompatibility and biodegradability combined with their high porosity and large surface area make this type of particle a very interesting substrate on the design of encapsulation platforms based on the LbL method. However, the large polydispersity and high potential of aggregation during the assembly process reduce the applicability of CaCO_3_ templates. On the other side, soft templates include different colloidal systems ranging from liposomes and vesicles to micro- and nano-gels, and from emulsion droplets to different types of cells. The main disadvantages of this type of template are related to their poor stability and elevated polydispersity. Furthermore, their handling is at many times difficult, making it necessary to use buffers and other special conditions. However, their utilization in applications requiring the interaction with biological matter can be a good choice due to their good biocompatibility and biodegradability [167].

The above discussion illustrates the existence of capsules based on the LbL assembly with a broad range of morphologies and dimensions, which in most of the cases can be modified almost at will to fulfill the requirements of their specific application.

### 3.2. Approaches for LbL Assembly on Colloidal Surfaces

The assembly of LbL polyelectrolyte films needs to consider that whereas inter-polyelectrolyte complexes formed in the bulk present thermodynamic stability, the assembly process of polyelectrolyte multilayers leads to the formation of kinetically arrested supramolecular structures [29,97,168,169]. This may be understood considering the release of a polyelectrolyte chain from the multilayer to the solution, with such processes being associated with an important gain on the entropy of the released chain (mainly the translational and conformational one) due to the enhanced mobility of the chain in solution with respect to the situation found in the multilayer [170].

**Figure 5:**
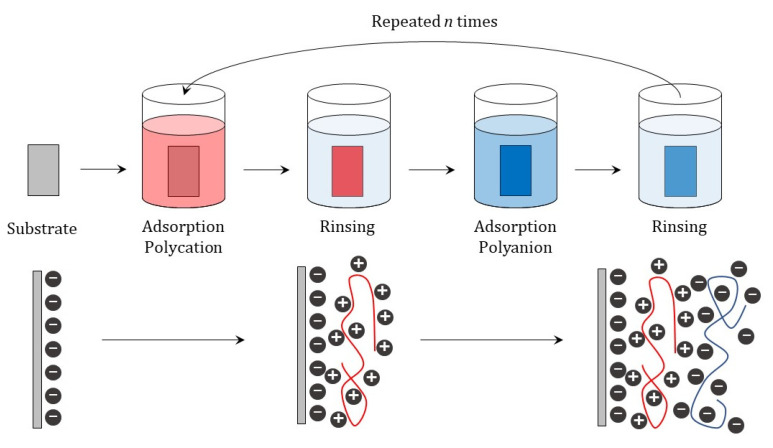
Sketch of the common methodology used for the fabrication of Layer-by-Layer (LbL) multilayers on a flat substrate by deposition for immersion.

The LbL approach is currently established as a simple and inexpensive methodology enabling the fabrication of multilayered structures [171,172]. This methodology has been applied on both flat substrates and colloidal templates, using commonly a diffusion-controlled kinetics to control the deposition of the building blocks on the surface [29]. It should be noted that most of the assembled LbL systems are obtained following a procedure that is reminiscent of that initially introduced by Decher et al. [173]. However, even though the physical bases of the assembly process are quite similar with independence of the used substrate, it is necessary to make some modifications on the experimental methodology to adapt the assembly procedure to the specific characteristics of the substrate, e.g., size, shape, morphology, or chemical nature. Thus, the assembly of layers of LbL films on a flat substrate has been commonly performed by simple immersion of the substrate into a solution containing the building block to be assembled, i.e., the so-called dipping deposition. In particular, the deposition of a multilayered film onto a flat substrate relies on the alternate immersion of the substrate into the layering solutions, with intermediate rinsing steps between two consecutive adsorption steps. These rinsing steps allow removing the material weakly adsorbed to the multilayer, avoiding any risk of cross-contamination of the assembled films due to the formation of aggregates for interaction in solution of the building blocks and their subsequent precipitation onto the multilayer (*Note: this rinsing step is critical when the assembly of polyelectrolyte layers is concerned, because the unbound polyelectrolyte molecules may be removed from the multilayers during the adsorption of an oppositely charged layer, resulting in the formation of inter-polyelectrolyte complexes in solution. These complexes can be deposited on the multilayered structure, affecting the structure and composition of the films*) [17,92,174]. Figure 5 shows a scheme of the common methodology used for the deposition of an LbL film by immersion of a flat substrate in solutions of polyelectrolytes bearing opposite charges.

It should be noted that one of the main drawbacks associated with the use of an immersive approached on the deposition of LbL films is associated with the long time required for the fabrication of each layer. This can be partially solved by adding to the layering solution an organic solvent, e.g., dimethylformamide or dimethylsulfoxide. This induces a dewetting process during the deposition process, which allows the elimination of the rinsing step and consequently increases the velocity of the assembly of LbL materials up to 30-fold [175,176]. Another alternative for fastening the deposition is to include a constant stirring of the solutions during the deposition, which allows limiting the deposition times to 10–20 s [177]. Furthermore, the spray-assisted and spin-assisted deposition methods have gained importance as alternatives to the dipping deposition because they allow a significant reduction on the fabrication time, increasing the homogeneity of the deposited films [178,179,180,181,182].

The use of the LbL approach on the fabrication of materials with a real application have made necessary to advance toward assembly strategies allowing for the scalability of the process from laboratory to the industrial level [183]. This has been possible by seeking methodologies enabling a reduction of the deposition time and a fine control of the film properties. This research has resulted in a broad range of methodological approaches, including the use of automated deposition machines and the introduction of non-conventional fabrication methodologies that are not limited only to the random transport of the materials from the solution, e.g., high-speed spinning, spraying, or fluidic and vacuum-based assembly [29,176]. In the following, we will highlight some of the currently used methodologies for the assembly of LbL films on colloidal templates. It should be noted that this section is not intended as a comprehensive revision of all the methodologies available for the assembly of LbL materials. For a most comprehensive analysis of the deposition methodologies, the reader is recommended to go through references [11,12,29,176,184].

**Figure 6:**
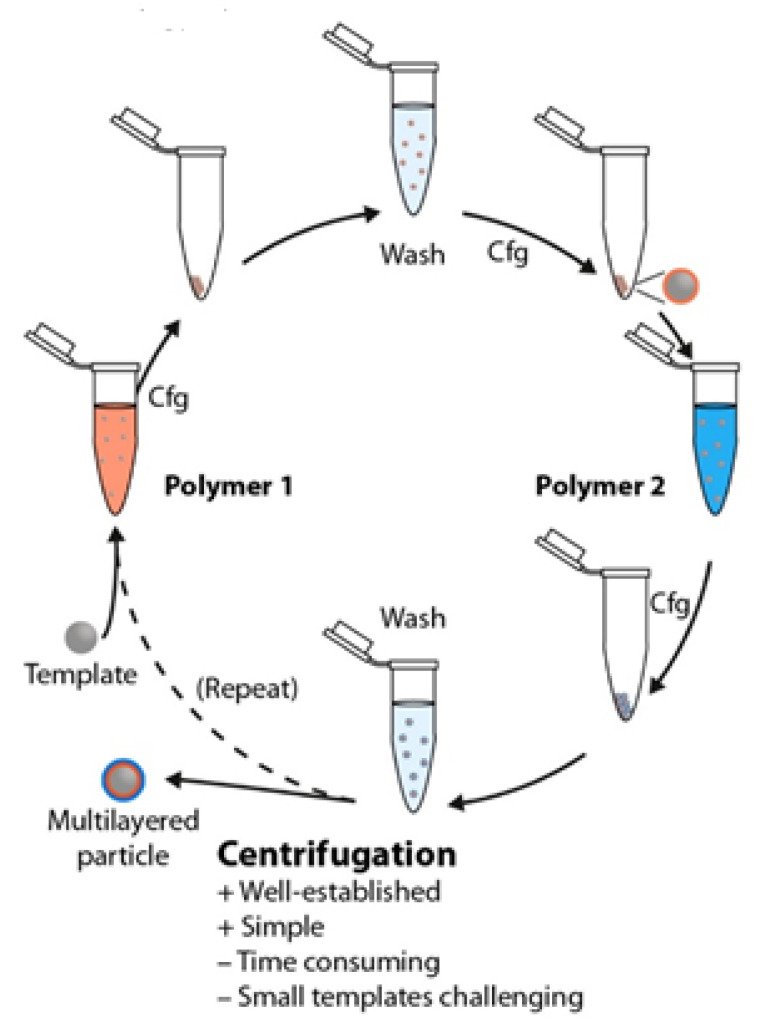
Sketch of the most frequent approach for the deposition of LbL multilayers onto colloidal particles. Reprinted from reference [184], Copyright (2014), with permission from the American Chemical Society.

The deposition by immersion in flat substrates appears as a rather easy approach on the fabrication of polyelectrolyte multilayers. However, the use of this approach for depositing multilayered films onto colloidal templates requires considering that colloidal particles are commonly dissolved or suspended in a solvent, generally water. This makes the assembly process tricky, because it needs to include a separation step between the deposition and washing. Thus, then, the excess of unbound materials is removed by pelleting the decorated colloids using commonly centrifugation/redispersion cycles on the washing steps when solid particles are used as a template [20,184,185,186,187,188,189]. Thus, once the adsorption of one layer is finished, the suspension containing the polymer-decorated particles is centrifuged, which results in their sedimentation at the bottom of the tube. This enables removing the supernatant where the excess of non-adsorbed material remains. Afterwards, the polymer-decorated particles are redispersed in the solvent, and the sedimentation/redispersion process is repeated (normally three times). The repetition of the sedimentation/redispersion process allows ensuring that the excess of non-adsorbed material is removed, resulting in a clean suspension of polymer-decorated particles that can be used as substrate for the deposition of a new layer following a similar sequence of adsorption–cleaning [190,191]. Figure 6 shows a sketch where the different steps involved in the deposition of polyelectrolyte multilayers onto a particulate template are displayed. This approach allows building both core-shell structures and a hollow capsule. On the fabrication of the latter, an additional step of dissolution of the template (sacrificial substrate) is required. This is generally done by a chemical treatment that depends on the specific chemical nature of the colloidal template, e.g., fluoride acid is used for SiO_2_ templates, diluted hydrochloride acid is used for melamine formaldehyde resins, and tetrahydrofuran is used for polystyrene latex particles [192].

The above-described methodology allows an easy building of polyelectrolyte multilayers onto colloidal microparticles. However, the separation of the supernatant and the polyelectrolyte-decorated particles by centrifugation (they require a high centrifugation speed, which can favor the aggregation of the polyelectrolyte-decorated particles) is more complicated when nano-sized colloids are chosen as the template. Furthermore, the use of centrifugation steps makes the automatization of the assembly process difficult. A possible solution to these problems is the use of the serum replacement method, which minimizes the aggregation of the obtained materials [193]. Another approach is the use of solvent-induced precipitation for the separation of the excess of non-adsorbed material [194]. These methods contribute to the increase of the velocity of the assembly process and increase the recovery yield (the use of centrifugation as the recovery approach results in a loosening of material and the subsequent decrease of the concentration in the successive cycles). The latter is very important in terms of a possible industrial scaling up of the assembly process, with the preparation of concentrated capsules suspensions being accounted for as one of the most important challenges for the assembly of materials by the LbL method. The use of tubular flow-type reactors has solved partially the problems associated with the scaling-up of the LbL fabrication. This approach results in the fabrication of capsules with a fixed number of layers following a continuous production procedure. However, this type of process may be affected by cross-contamination because a small amount of the polyelectrolyte forming the last deposited layer is always retained in the medium after each deposition step, which can result in the precipitation of inter-polyelectrolyte complexes [195].

An alternative to the centrifugation is the use of a magnet for separating the substrates from the excess of non-adsorbing molecules [196]. This has been used for the separation of emulsion droplets, which cannot be easily pelleted using centrifugation. This process requires the loading of the droplets with magnetic nanoparticles [197]. This approach has been also used for the separation of solid particles. In such case, the substrate used as template needs to have the inclusion of a magnetic material [198].

It should be noted that in most of the cases, the LbL assembly on colloidal templates is performed using highly concentrated polymer solutions (many times the concentration required for coating the particles) [187,199], which, as was aforementioned, makes it necessary to introduce strategies for separating the polyelectrolyte-decorated colloidal particles from the excess of non-adsorbing material. However, the washing/separation steps can be avoided by adding just the amount of polyelectrolyte necessary for saturating the colloidal particles. The optimization of this process requires a careful observation of the zeta potential of the dispersion for avoiding the aggregation. Furthermore, the sonication of the dispersion during the layer deposition can also contribute to minimizing the aggregation phenomena [136,200,201,202]. The use of the saturation method for LbL assembly allows an increase of the velocity of the process by a factor of 3 [201,203].

The centrifugation can be a serious problem when particles lighter than water (e.g., emulsion drops, vesicles, or liposomes) are used as templates. This has made it necessary to adapt the methodology of the LbL fabrication to the specific characteristics of the templates. The use of creaming/skimming cycles to substitute the centrifugation/redispersion one is a promising alternative for separation of the excess of non-adsorbed material when droplets of oil in water emulsions are used as a template. This is possible because the droplets are generally lighter than water and can float in the polymer solution [204,205]. It is worth mentioning that the centrifugation can be useful to force a faster creaming process when emulsion droplets are used as the template [206,207,208]. The use of vesicles or liposomes as a template for LbL assembly requires a very complex process involving up to three different steps for each deposited bilayer [209]. First, the solution containing the first layer is added to a diluted suspension containing vesicles/liposomes. After the adsorption of the first layer, the solution of the material forming the second layer is added to the dispersion containing the polyelectrolyte-decorated vesicles/liposomes and an excess of the polyelectrolyte forming the first layer. This results in the deposition of the second layer and the formation of inter-polyelectrolyte complexes. This inter-polyelectrolyte can be sedimented from the dispersion containing the polyelectrolyte-decorated vesicles/liposomes through centrifugation in the last step of the process, with the use of centrifugation for the separation being possible because the formation of the polyelectrolyte shell provides stability to the vesicles/liposomes, hindering the aggregation and fusion phenomena. Once the first bilayer is deposited, the process can continue by repeating the above commented procedure up to depositing the desired number of layers. However, it should be noted that this procedure does not allow depositing more than five to six bilayers, because the formation of inter-polyelectrolyte aggregates that can form supramolecular aggregated with the polyelectrolyte-decorated vesicles/liposomes and the centrifugation process result in a 5% reduction of the total number of vesicles/liposomes per each deposited bilayer [209].

Another very interesting approach for avoiding the use of complicated separation steps relies on fixing the colloidal template by using immobilization agents, e.g., agarose hydrogel. This makes it possible to consider the collected particles as a planar substrate, allowing the use of conventional dipping for forming the LbL film onto the colloidal particles. Once the required number of layers has been deposited onto the colloid, it is possible to erode the agarose matrix by heating at 37 °C. Then, the agarose and the polyelectrolyte-decorated colloids are separated by at least three cycles of centrifugation/re-dispersion [210]. The use of the above discussed approach has allowed the use of robotic dipping for the assembly of LbL films on colloidal templates [210]. It should be noted that the automatization is one of the current challenges of the LbL method; several approaches exist toward the automatization of LbL deposition on colloidal templates [29,210,211,212]. Peiffre et al. [213] designed a computer-controlled device that enables the fabrication of about 1000 layers on particles of 100 μm in diameter. Another very interesting approach for automatizing the assembly on non-planar substrates involves the filling of pores with polyelectrolyte and colloid under pressure application. Thus, it is possible to fabricate even polymer nanotubes upon the dissolution of the substrate [214].

Another approach gaining interest in current years is the use of microfluidic devices. Thus, it is possible to fabricate LbL films on the channels and colloidal templates placed in the channels [212,215,216,217]. In particular, the use of microfluidic devices allows a minimization of the aggregation processes during the assembly [218,219,220,221,222]. There are many approaches on the use of microfluidic devices for the LbL assembly. The most common is based on the application of pressure or vacuum to drive the sequential displacement of the polymer and washing solutions within the microfluidic chips [120,223,224]. This relies on coating liquid particles by flowing alternatively the solutions for layer deposition and washing perpendicularly to the particle flow stream [219]. Another alternative for the deposition using microfluidic methodologies for LbL deposition involves an alternative exposure of the colloidal templates to the polyelectrolyte and washing streams [222].

The use of a fluidized bed for the layering process is also a very promising alternative for coating with LbL films on particulate substrates [225]. This methodology takes advantage of the upward force of the washing or polymer solutions that counteract the gravitational forces that push the particle to the sedimentation. Thus, the particles are lifted, forming a fluidized bed allowing the fabrication of LbL film on substrates up to 3 μm by modifying the bed geometry, which also allows controlling the permeability of the resultant multilayered films [226]. It is true that the use of microfluidic for LbL assembly on colloidal templates opens new perspectives. However, their implementation is limited, because it needed an optimization of the methodology in each particular case, and the instrumentation is expensive [227].

The above discussion shows that the LbL approach offers many opportunities in the assembly of nanostructured materials on colloidal templates using for such an approach different fabrication methodologies, which opens many opportunities for both fundamental and applied sciences. However, any program aimed at the development of the potential applications of LbL materials requires deeper understanding of the most fundamental aspects associated with the film formation at the molecular scale and the properties of the built materials, with two characteristics of the multilayers being essential: (i) polyelectrolyte multilayers are highly hydrated systems and contain counterions, and (ii) the adsorption of polyelectrolyte layers is almost irreversible.

## 4. LbL Multilayers on Liposomes

Liposomes are small spherical vesicles based on a lipid bilayer and an internal cavity commonly filled with water [228]. This makes liposomes a very interesting biocompatible tool on the fabrication of platforms for the encapsulation and delivery of hydrophilic and/or hydrophobic molecules with technological interest [229]. Furthermore, the encapsulation within liposomes of drugs allows increasing the efficacy and therapeutic index of the drugs, whereas their toxicity is reduced [230]. However, the applications of conventional liposomes appear very limited due to their low stability in physiological conditions, presenting a high tendency to degradation or aggregation and fusion, and the limited efficiency of their adsorption through tissues. In the last few years, researchers have tested different approaches that allow enlarging the life of liposomes in the bloodstream, with their coating with a single poly(ethylene glycol) to form the so-called stealth liposomes being accounted among the most extended. This increases the hydrophilicity of the liposome, and reduces the biofouling phenomena; i.e., the adsorption of plasmatic proteins and lipoproteins [231,232]. Other polymers used for coating liposomes with a single layer are chitosan or poly(vinyl alcohol) [233,234,235].

It should be noted that together with the enlarging of the life of the liposomes, their protection against aggressive environments, such as appearing in many physiological media (e.g., stomach) is currently a challenge. Chen and Santore demonstrated that the stability of liposomes can be significantly enhanced by forming a mixed membrane formed by phospholipid and a grafter copolymer [236]. Other possibilities include (i) the decoration of the liposome surface with moieties that enable the recognition of specific targets in some cells; (ii) increasing the number of possible drugs and the amount stored in the membrane, or (ii) tuning the delivery rate by coating the liposomes with other motives, such as polyelectrolytes, nanoparticles, or smaller liposomes [237].

**Figure 7:**
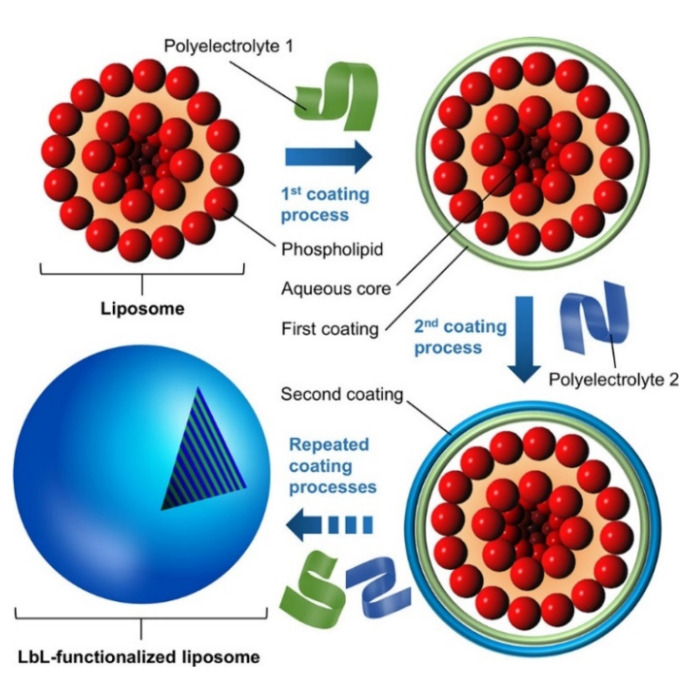
Sketch of the LbL approach for the fabrication of different LbL decorated liposomes. Reprinted with permission from reference [238]. Copyright (2020), with permission from the American Chemical Society.

The use of LbL layers for coating liposomes and manufacturing core–shell supramolecular systems has become a very promising tool in current years for broaden the application fields of liposomes [238,239] and improving their therapeutic efficiency [240,241,242]. Cuomo et al. [26,243,244,245] and Fukui et al. [246,247] showed more than 10 years ago that the use of the LbL method for coating liposomes with polyelectrolyte multilayers may be a good opportunity for designing stable drug delivery platforms with tunable release profiles. Furthermore, the use of the LbL method also makes the construction of different complex hierarchical multi capsules possible, as evidenced the works by the group of Caruso [74,75,76,77]. Figure 7 shows a scheme of the procedure used for the fabrication of liposomes coated with LbL films.

The coating of liposomes with LbL films prevents their aggregation by the electrostatic repulsion associated with the surface charge provided by the polyelectrolyte layers. Furthermore, it was found that the efficiency of liposomes transport in physiological media and their adsorption through tissues are also strongly enhanced by decoration with LbL films [248]. Another important advantage of the use of liposomes decorated with layers deposited using the LbL method is the possibility to introduce controlled release capacity in the supramolecular system [249,250,251] and the inclusion of an additional hydrophilic environment within the deposited layers to increase the payload of active molecules [239]. This was demonstrated by Haidar el al. [239] upon coating cationic liposomes with alternate layers of alginate (ALG) and chitosan (CHI). They found that the amount of bovine serum albumin (BSA) may be significantly enhanced by the increase of the number of deposited bilayers due to the embedding of additional protein molecules within the polyelectrolyte layers. This agrees with the results by Pereira-Parchen et al. [252] for the encapsulation of epidermal growth factor, with a 3-fold increase of the encapsulation efficiency for decorated liposomes in relation to the bare one.

Moreover, the addition of polyelectrolyte layers results in a more sustained release of the encapsulated proteins than that found in bare liposome, with the latter showing a burst release of the 60% of the cargo within the first 3 days [239]. Therefore, the LbL films improve the performance of bare liposomes from two perspectives: (i) increasing the encapsulation efficiency, and (ii) acting as a barrier against the encapsulated molecule leakage. This agrees with the finding by Liu et al. [253] for negatively charged liposomes decorated with multilayers of lactoferrin (LF) and BSA. The coating of the liposomes with the multilayered film provides them with significant stability against heating treatment, pH changes, and long-term storage, which is increased with the number of deposited bilayers. Similar results were found for lecithin liposomes coated by alternated layers of CHI and dextran sulfate (DXS) [254]. Furthermore, the coated liposomes are less damaged by gastrointestinal fluid than the nude liposomes used as template, also having an improved release profile of encapsulated cargoes. This improved release was also reported for quercetin-loaded liposomes coated by up to five bilayers formed by CHI and HA, with a more sustained release emerging as the number of deposited layers [255]. Furthermore, the multilayered film ensures a better stability of the liposomes against the disruption of their membranes by detergent (Triton X-100), and it enhances the permeation ability of the liposomes through the skin barrier. It should be noted that the formation of LbL layers on the liposomes surface may also provide a partial protection against the action of natural phospholipases if a minimal thickness of the coating is obtained [256].

**Figure 8:**
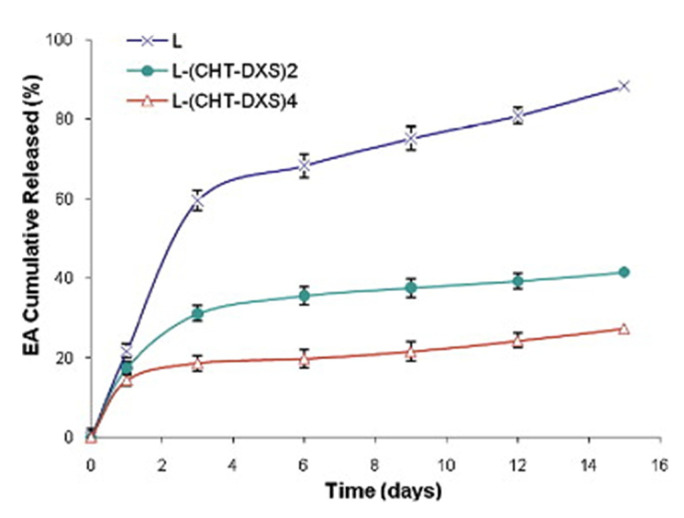
Release profiles for ellagic acid from bare liposomes (L) and biopolymer-coated liposomes with two and four bilayers (CHT: chitosan and DXS: dextran sulfate). Data adapted from reference [254], Copyright (2010), with permission from Elsevier.

Madrigal-Carballo et al. [254] performed a systematic evaluation of the release profile of ellagic acid from bare and polyelectrolyte multilayer decorated liposomes, and they found a transition from a burst release in the former to a more sustained release with the increase of the number of deposited bilayers (see Figure 8). In fact, for bare liposomes, 70% of the load molecules are released during the first week, whereas in formulations with two bilayers of CHI and DXS, the released amount is reduced to around 35%, and in those with four bilayers, the released amount is reduced up to 2-fold. This is explained considering that the increase of the number of bilayers results in an increase of the ionic cross-linking, leading to the formation of a densely packed polymer network, which reduces the release rate from the liposomal core. It should be noted that even LbL coating layers avoid the leakage of cargoes encapsulated from the liposomes. This protection is not complete, and a certain degree of leakage appears as the temperature increases. However, the leakage is reduced up to 20% in relation to nude liposomes.

Zhang et al. [257] optimized the loading of insulin in nanoliposomes (diameter around 100 nm) by its deposition as polyanionic layer between positively charged CHI layers (five bilayers). This technology allows preparing nanoliposomes formulations containing 10 wt % of insulin. This content is 10-fold higher than the maximum amount of insulin, which can be included within the inner aqueous core of bare nanoliposomes and other capsules and may be increased by depositing additional insulin–chitosan bilayers. Furthermore, the use of capping chitosan layers facilitates the cellular uptake and transport of nanoliposomes formulation without compromising the insulin activity, and it ensures the protection of insulin during intestinal penetration.

Most of the studies about LbL-decorated liposomes have been focused on the fabrication of barrier layers, which avoid the leakage of cargoes from the core and ensure a controlled release. However, less attention has been paid to the fabrication of engineered multi-functional supramolecular systems to targeted delivery and facilitated site-specific triggered release [249,258]. Hashemi et al. [259] reported the fabrication of a dual chemo-photothermal therapeutic platform with encapsulated doxorubicin formed by alternated layers of graphene oxide (GO, negatively charged macroion) and poly(L-lysine) with grafted graphene oxide nanosheets (PLLgGO) deposited on positively charged liposomes. The deposition of the multilayers was found to be governed by electrostatic interactions, and it was dependent on the concentrations of the building blocks and the GO or PLLgGO-to-liposome mass ratio. The higher the mass ratio, the lower the zeta potential and the higher the average size increased. However, the deposition of a maximum of two bilayers on the nude liposome was only possible due to the emergence of aggregation phenomena. The liposomes coated by (GO-PLLgGO)_n_ multilayers were found to present good thermo- (GO has an ability for heat generation upon light adsorption) and pH-responsiveness, which were exploited by the controlled release of the encapsulated doxorubicin. Furthermore, the presence of PLLgGO may facilitate the fabrication of effective binding platforms for active targeting.

**Figure 9:**
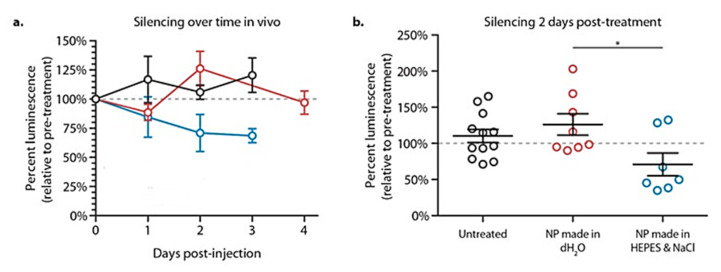
(**a**) Temporal evolution of the gene silencing effect of liposomes loaded with a 21-base-pair locked nucleic acid siRNA analogue on the human ovarian cancer cell line OVCAR8 depending on their preparation protocol: untreated (black symbols), assembled in deuterated water (red symbols) and assembled in HEPES and NaCl (blue symbols9 (**b**) Gene silencing effect of liposomes loaded with 21-base-pair locked nucleic acid siRNA analogue on the human ovarian cancer cell line OVCAR8 depending on their preparation protocol after two days of their injection. Reprinted with permission from reference [260]. Copyright (2019), with permission from American Chemical Society.

It is well known that the assembly conditions of LbL films have a very critical impact on the physicochemical properties and structure of the assembled material [11,12,97]. This is even more critical when the fabrication of LbL-decorated liposomes for drug delivery purposes are fabricated, with an optimal film formation making it necessary to tune the solution conditions during both the layer deposition and the cleaning steps. This was clear from the work by Correa et al. [260]. They found that the liposomes loaded with a 21-base-pair locked nucleic acid siRNA analogue prepared under optimized assembly conditions (layered in solutions containing 25 mM NaCl and 20 mM HEPES, and cleaned in deionized water, DI H_2_O) allow an effective gene silencing on the human ovarian cancer cell line OVCAR8. The silencing effect was found to be more limited when the assembly of the LbL films was performed only using DI H_2_O during assembly and cleaning steps. Figure 9 shows the different gene silencing effect of liposomes loaded with 21-base-pair locked nucleic acid siRNA analogue on the human ovarian cancer cell line OVCAR8 depending on their preparation protocol.

The optimization of the coating of liposomes with LbL multilayers was also analyzed by Liu et al. [261]. They prepared liposomes coated by alternated layers of CHI and ALG for fabricating drug delivery systems, and they found that the concentrations of both polyelectrolytes play a very significant role in the preparation of the formulation, with the concentration of CHI and ALG appearing as very important parameters for controlling the average diameter, zeta potential, and coating efficiency. In fact, it was reported that high polyelectrolyte concentrations stimulated the flocculation of the coated liposomes, and hence, intermediate concentrations were chosen, which results in formulations with a high resistance against destabilization processes. The importance of the control of the aggregation mechanism of polyelectrolyte-decorated liposomes by a careful selection of the concentration of the layering solution was also reported by Madrigal-Carballo et al. [254] and Hermal et al. [256] in liposomes coated by CHI and DXS and PLL and poly(glutamic acid) (PGA), respectively. Furthermore, the coating process ensures the protection of the liposomal core against pH changes, whereas the polyelectrolyte shell undergoes a swelling process with the increase of the pH. The latter is related to a decrease of the ionic cross-linking between the polyelectrolyte chains and the dissolution of ALG for pH > 5. However, the integrity of the liposomes remains due to the collapse of insoluble CHI chains on the liposomes, which results in a new reduction of the average size of the polyelectrolyte-decorated liposomes. This agrees with the general picture showing that the use of LbL films for coating the liposomes enhances their stability. However, the increase of liposome stability depends on both the number of deposited polyelectrolyte layers and the charge density of the liposomal surface, which indicates the important role of the electrostatic interactions as driving force for the stabilization [262]. This is compatible with the finding by Pereira-Parchen et al. [252] for the release of epidermal growth factor from liposomes decorated with (CHI-ALG)_n_ multilayers. They reported that the drug release is completely hindered for physiological values of the pH (around 7.2) due to the compact character of the CHI layers, whereas the swelling of the multilayer with the decrease of the pH favors the release of the encapsulated drug. Further evidence related to the role of the electrostatic on the stabilization of liposomes with LbL films was reported by Eivazi et al. [263]. They studied the protection of soft cationic liposomes formed with didodecyldimethylammonium bromide by coating using two cellulose derived polymers (anionic carboxymethylcellulose and cationic quaternized hydroxyethylcellulose ethoxylate), and they found that the electrostatic interactions appear as the essential driving force for the assembly of the LbL layers on the liposomes, with their modulation enabling control of the size and net charge of the core-shell particles. These particles were found to have a shape that is reminiscent of the initial template.

The impact of the electrostatic interactions in LbL-decorated liposomes goes beyond its impact on the protection of encapsulated drugs and the integrity of the bare liposomes. The surface charge of the bare liposomes should be chosen in such a way that ensures a strong electrostatic binding between the polyelectrolyte chains and the first deposited layers [113,168,264,265,266,267]. This was clear from the studies by Angelini et al. [262] in which an analysis of the stability against the detergent of the liposomes coated with LbL layers was performed. They found that the interaction of charged liposomes with strongly charged polyelectrolytes results in the formation of regular and homogenous layers with a reduced porosity, and hence, the stability of the coated liposomes is significantly enhanced. On the other side, the interaction of polyelectrolytes with poorly charged liposomes leads to a non-homogeneous deposition of the first layer, and the stability of the decorated liposomes is not significantly enhanced upon the LbL coating [268,269,270,271]. Furthermore, the control of the charge nature and density of the last layer can influence the uptake of the coated liposomes through different tissues [272]. This is also related to the surface roughness of the LbL-decorated liposomes, the smaller the surface roughness, the higher the uptake of the decorated liposomes by cells and tissues. It should be noted that the roughness of the LbL films can be modified almost at will by changing the environment, as was demonstrated by Verma et al. [273] in liposomes coated by multilayers formed by ALG and chitosan conjugated to Vitamin B_12_. As a result, the adhesion force of the external surface of the multilayer coating the liposomes may impact decisively on their efficiency as delivery systems of therapeutic and vaccines.

On the basis of the above discussion, the optimization of the assembly of LbL films on liposomes is very important and should consider the following aspects: (i) the saturation of the surface of the liposomes is not easy because of the absence of a homogenous distribution of charge along the polymer chains and the difficulties associated with a complete binding between monomers of adjacent layers as a result of the steric hindrance. This can increase the heterogeneity and roughness of the multilayers; (ii) there are difficulties associated with the exact matching in the charge density of assembled polyelectrolytes, which is an important requisite for the LbL deposition; (iii) the use of polymer in excess to ensure a complete coverage may result in the existence of unattached polymer chains remaining in solution, which may induce depletion flocculation; (iv) the increase of the size of the decorated liposomes with each deposition cycle makes it necessary to increase the concentration required to cover their surface, resulting in an increase of the complexity of the morphology of the polymer layers, and (v) Lastly, the existence of other interactions beyond those of electrostatic origin need to be considered as well [274].

It is true that the optimization of the assembly conditions appears as essential for controlling the efficacy of formulations. However, another very important aspect to analyze when liposomes coated by LbL films are considered as delivery platforms is related to the effect of the cargoes on the stability of the supramolecular material. This aspect was analyzed by Madrigal-Carballo et al. [254] in liposomes coated by CHI and DXS loaded by ellagic acid, and they found that the introduction of the cargo affects neither the average size of the decorated-liposomes nor their surface charge. This appears as a signature of the negligible impact of the encapsulated molecule on the stability of the formulation.

## 5. LbL Multilayers on Emulsion Droplets

Another feasible approach for the fabrication of encapsulation platforms for oil-soluble compounds is the use of the liquid droplets of oil in water (o/w) emulsions as a template for the assembly of LbL multilayers. The coating of emulsions droplets with LbL films enables including different functionalities and molecules, both inside the emulsion volume and in the shell. Furthermore, the LbL coating allows introducing controlled release abilities to the capsules, which is not possible in most cases when bare emulsions are considered. It should be noted that capsules obtained by coating emulsions are not as robust as those obtained using a hard colloidal particle as template [204]. However, these capsules appear more stable than bare emulsions due to a combination of the thickness, electrical charge, and packing density of the deposited layers [25]. This can broaden the application range of LbL polyelectrolyte capsule in cosmetics, food science, or medicine [202,275,276,277,278,279,280,281,282].

There are several preparation strategies, which enable the preparation of LbL coatings on emulsions droplets and minimize their aggregation as a result of the excess of free polyelectrolyte chains [193,283,284,285,286], with the assembly of the macromolecular layers on the oil droplets being performed once the bare emulsion droplets are stabilized for the formation of a layer of the emulsifier at the oil/water interface [287]. This emulsifier can present different nature, e.g., amphiphilic polymer [288], proteins [289,290], polysaccharides [291], or phospholipids [292]. Furthermore, the possible fabrication of multilayered films on the emulsion droplets is only possible when complementary interactions appear between the emulsifier layer and the building block used for assembly of the first layer [293]. The adsorption of the following layers is usually performed trying to reduce the excess of non-adsorbed molecules through one of the following routes:

- Saturation method. It involves the addition of a very limited amount of polyelectrolyte to the emulsion to ensure that almost all the molecules adsorb on the droplet surface, and only a reduced number of chains remain free in solution. It is convenient when the saturation method is used to monitor the zeta potential of the emulsions during the mixing with polyelectrolyte. Thus, it is possible to determine easily the saturation point by the polymer concentration in which the onset on the plateau of the dependence of the zeta potential on the polymer concentration is reached. This value is generally close to that corresponding to the zeta potential of the polyelectrolyte [202,284].
- Centrifugation method. It relies on adding an excess of polyelectrolyte to the emulsion, which ensures an optimal coating of the droplets. The decorated droplets are settled down by centrifugation, separation of the supernatant containing the excess of polyelectrolyte, and finally re-suspension in an appropriate aqueous solution. The repetition of this procedure several times ensures a complete removal of the free polyelectrolyte before the addition of the next layer. The main problem of this approach is related with the emergence of aggregation phenomena because the decorated droplets are deformed and forced to be very close during the centrifugation, which may result in coalescence phenomena. Furthermore, the centrifugation is difficult when emulsions with small droplets are considered due to the small density difference between the droplets and the continuous phase.
- Filtration. This method also relies on the addition of an excess of polyelectrolyte. However, the separation of the decorated droplets and the non-adsorbed molecules is performed using a forced filtration using a membrane (cut-off in the range 50–100 nm), which allows the polyelectrolyte chains to pass through, but not the coated droplets. Simultaneously to the filtration process, a polymer-free aqueous solution is added to the colloidal suspension, ensuring that its concentration remains unchanged.

It should be noted that independently of the method used for the preparation of the emulsions decorated with polyelectrolyte multilayers, a careful control of the composition and preparation conditions is necessary to ensure that stable dispersions are obtained. Thus, it is necessary to ensure that the amount of polyelectrolyte added is high enough to ensure a complete coating of the droplet, but at the same time, it is not recommended to introduce a large excess of polyelectrolyte for avoiding depletion flocculation processes. This means that the fabrication of emulsions decorated with LbL films is only possible when the adsorption of the polyelectrolyte molecules on the droplets is faster than the droplet–droplet collision [294].

**Figure 10:**
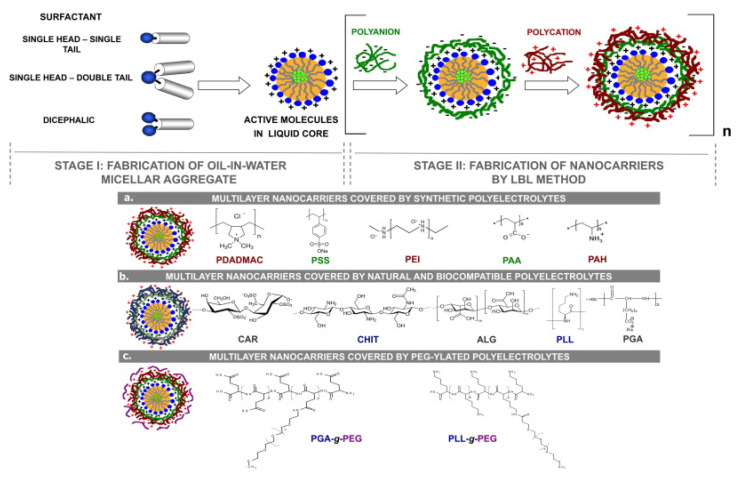
Sketch of the LbL approach for the fabrication of different nanocarriers using multilayers. Reprinted with permission from reference [284]. Copyright (2015), with permission from Elsevier.

One of the first attempts to apply LbL films for increase the stability of o/w emulsions was performed by Grigoriev et al. [204]. They prepared emulsions of dodecane and chloroform in water stabilized by didodecyldimethylammonium bromide (DODAB), which were coated by the sequential adsorption of PSS and PDADMAC or PAH layers, and they demonstrated that o/w droplets were a good substrate for LbL deposition. Furthermore, by inclusion of an oil-soluble dye on the inner region of the LbL decorated droplets, it was demonstrated that the oil phase can be either the core of the capsule or the cargo, with the latter enabling controlled release upon demand by modification of the media properties. Figure 10 displays a sketch of the common process used for the fabrication of emulsions droplets coated with LbL layers together with the most common types of decorated emulsions fabricated using the LbL approach and the polyelectrolytes used for such fabrication approaches.

The use of LbL for coating emulsion droplets to protect different hydrophobic molecules in the oil core is widespread. Szczepanowicz et al. [295] explored the protection of curcumin by their encapsulation within the oil phase of chloroform in water emulsions stabilized by aerosol OT and coated combining PLL and PGA, and they found that the inclusion of the curcumin within the decorated emulsion droplets results in an enhanced activity of curcumin against H_2_O_2_-induced cell damage. This suggests that emulsions decorated with LbL films may be a very promising alternative for the fabrication of drug delivery platforms.

**Figure 11:**
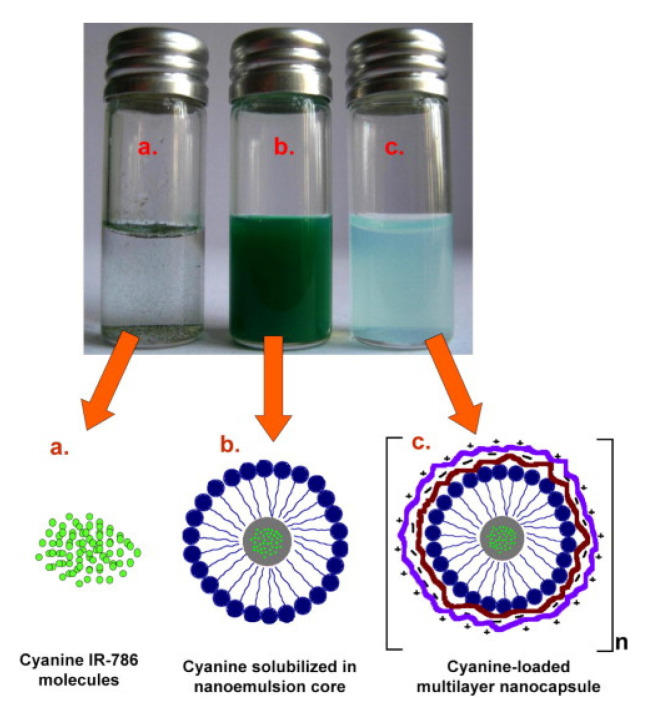
Cyanine IR-786 (**a**) dispersed in water, (**b**) solubilized in bare nanoemulsion, and (**c**) encapsulated in nanoemulsion coated by (PSS-PDADMAC)_4.5_. Reprinted with permission from reference [297]. Copyright (2012), with permission from Elsevier.

Bazylinska et al. [296] fabricated nanoemulsions coated with (PSS-PDADMAC)_n_ multilayers for the encapsulation of three dyes: cyanine IR-786, cyanine IR-780, and Oil Red O. They found that LbL-coated nanoemulsions were very useful tools for improving the dispersion of insoluble dye in water, with the highest retention of dyes (almost 95%) being found after only the deposition of four layers. Figure 11 shows an image where a dispersion of cyanine in water, bare, and LbL-coated nanoemulsions is shown [297]. Furthermore, the coated nanoemulsions with the encapsulated cargo presented a good stability upon storage during 21 days in dark conditions without evidences of photodegradation. The analysis of the assembly process of the multilayered films on the bare emulsion pointed out that a strong binding of the initial anionic layer to the surfactant layer may play a central role on the prevention of the release of the encapsulated dyes. Bazylinska et al. [296] also studied the release of the encapsulated dyes from the decorated nanoemulsions as a response to a change of the environmental conditions, and they found that the multilayered coating leads to a reduction of the typical burst release found in bare nanoemulsions, enabling a more sustained release. The studies on the dye release indicate that the stronger the binding of the first polyelectrolyte layer to the surface on the emulsion droplet, the better the ability for the sustained release.

The release of the encapsulated molecules is not only associated with the interaction between the surfactant layer and the first polyelectrolyte layer, with the interaction of the surfactant layer with the encapsulated molecules playing a non-negligible role. Bazylinska et al. [298] studied the release profiles of cyanine IR-786 from nanoemulsions stabilized with two different dicephalic-type surfactants, the cationic, *N,N*-bis[3,3′-(trimethylammonio)propyl]dodecanamide dimethylsulfate and the anionic, disodium *N*-dodecyliminodiacetatesurfactants, and coated with multilayers formed by PLL and λ-carragen (CAR). They found that the leakage of the dye from nanoemulsion stabilized by the anionic surfactant was three times faster than from those stabilized with the cationic system. This was assumed to result from a partial neutralization of the charge by the positive charge of the encapsulated drug, which favors the leakage from anionic nanoemulsion. On the other side, the encapsulation is more favorable when nanoemulsions by the cationic surfactant are considered as a result of the repulsive electrostatic interactions between species of the same type of charge.

Bazylinska et al. [297] exploited the above nanoemulsions as delivery platforms of cyanine IR-786 (photosensitizer) to human breast carcinoma cells for photodynamic therapy. The encapsulation process results in protection of the cyanine IR-786 against the photobleaching, which is very important for its photodynamic efficiency. The results evidenced that the viability of the carcinogenic cell may be reduced significantly upon irradiation when the capping layer was positively charged, which is explained considering a better cell penetration for capsules with positive surface charge [299]. Furthermore, the study by Bazylinska et al. [297] suggests that it is necessary to optimize the number of layers for ensuring a good protection of the actives without enlarging the release process beyond time-scales far from those involved in practical purposes. Thus, the number of deposited layers forming the coating should be high enough to avoid a burst release but not so high to hinder the permeability of the encapsulated molecules.

McClements et al. [300] evidenced that the number of deposited layers and their packing may be essential in the protection of the cargoes included within the oil phase, with the digestion of lipids included in the oil phase of emulsion as result of lipase activity being significantly hindered with the increase of the number of deposited layers. Thus, a total digestion of the lipids was found for uncoated emulsions after 30 min of lipase action, whereas this digestion is 2.5 fold reduced upon coating of the emulsions droplets with a single layer. This was associated with the formation of a dense layer, which minimizes the access of the lipase to the core of the emulsion, and thereby, the digestion is retarded. The formation of thick and dense layers appears as essential for the protection of the molecules contained on the emulsion core, as evidenced by the results by Lomova et al. [301]. They studied emulsions coated with different numbers of layers of multilayers formed by poly(L-arginine) (PLARG) and tannic acid (TA) or dextran (DX), and they found that the stability of the oil phase against oxidation was not enhanced upon coating with the multilayered films. This was attributed to the small thickness of the deposited shells.

The above discussion pointed out that the enhanced stability of LbL-decorated emulsions is possible by the steric hindrance and the electrostatic repulsion associated with the formation of LbL layers, which prevents the approaching of the oil droplets, and hence, the instability caused by depletion–flocculation is minimized. Furthermore, as was evidenced by the work by Azarikia and Abbasi [302], the increase of the oil concentration also reduces the instability of the emulsions as a result of the high dispersion viscosity, which retards the droplet motion and consequently their collision. It should be noted that the role of the electrical properties of the outer layer on the physical stability of emulsion droplets is very important. Sadovoy et al. [287] encapsulated eight volatile fragrances in emulsions decorated with multilayers of BSA and TA, and they found that the coating procedure provides a good protection of the fragrance against evaporation upon storage during two months. Furthermore, the multilayered films were able to reduce the evaporation even upon exposure of the dispersion to open air at 40ºC. Furthermore, it was found that the higher the hydrophobicity of the fragrances, the higher the encapsulation yield. This is explained considering the partition of the fragrances between the aqueous phase and the interior of the LbL capsule during the coating procedure.

**Figure 12:**
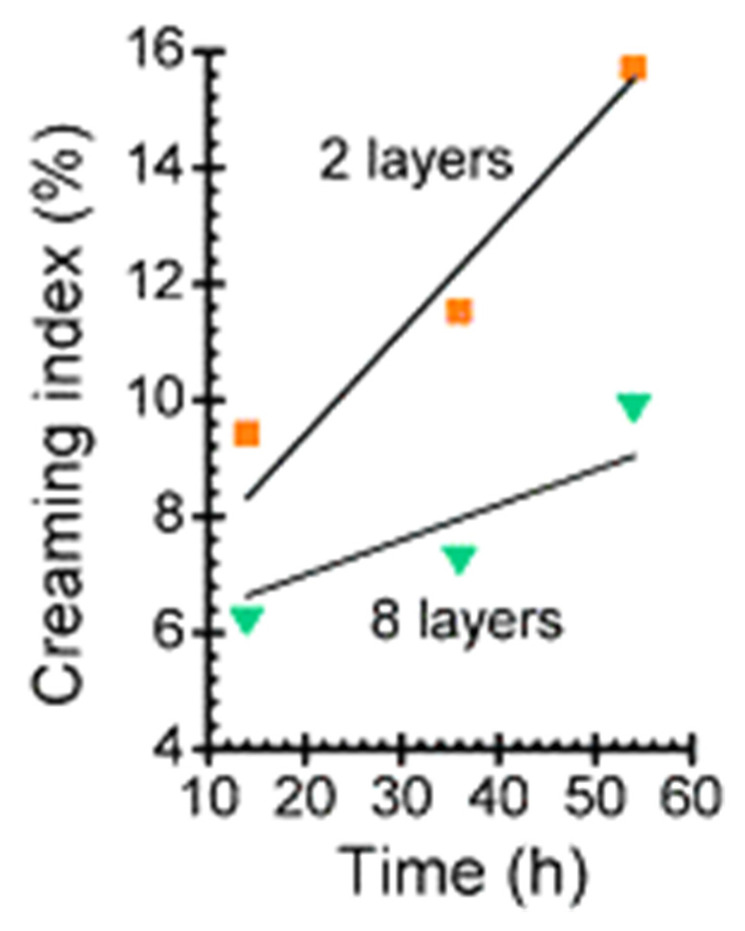
Time evolution of the creaming index for emulsions coated with different numbers of PSS and poly(allylamine hydrochloride) (PAH) layers. Reprinted with permission from reference [290]. Copyright (2011) with permission from PCCP Owner Societies.

McClemens et al. [300] studied the stabilization of emulsions droplets with alternate layers of positive CHI and negative β-lactoglobulin, and they found a strong dependence of the stability of the emulsions on the nature of the last layer and their charge. Thus, emulsions capped with β-lactoglobulin change their surface charge from positive to negative with the increase of the pH of the aqueous solution, and hence, they undergo destabilization at intermediate pH due to their low net charge. On the other side, emulsions capped with CHI present stability for pH < 6 due to the positive charge of CHI, and they are highly unstable for neutral and basic pH due to the limited charge of the CHI layer. Other examples of the effect of the net charge of the capping layer are found for emulsions containing pectin or ALG layers that undergo destabilization for pH < 3 due to the reduced negative charge of the biopolymers. However, the increase of the pH enhances the stability of emulsions capped with ALG or pectin due to the high negative charge of these polyanions [300]. Deepening the understanding of the stabilizing role of polyelectrolyte multilayers on emulsions droplets, Sadovoy et al. [290] analyzed by combination of theoretical predictions and experiments the gravitational separation profiles of coated emulsion droplets and found that such profiles depend on the shell thickness; the higher the shell thickness, i.e., the greater the number of deposited layers, the faster the creaming. Figure 12 shows the time evolution of the creaming index of emulsions coated with multilayers of PSS and PAH including different numbers of layers.

Lomova et al. [301] explored the stability against creaming of linseed oil stabilized by BSA and coated with multilayers formed by PLARG and TA or DX. For such purposes, they centrifugated emulsions aged during the 15 days and found that coated emulsions were stable without any evidence of phase separation. These results confirm that the deposition of multilayered films is a good methodology for improving the efficiency of emulsions as cargo carriers.

The ability of emulsions for encapsulating different types of cargoes also allows including colloidal objects, which can help drive the formulations to specific targets or stimulate a controlled release. This was explored by Mu et al. [197]. They prepared o/w emulsions loaded with Fe_3_O_4_ and a drug (dipyrimadole), which were coated by the deposition of oligochitosan (OCHI) and ALG layers. These capsules can be directly targeted by the application of magnetic fields, presenting a release profile easily tunable by pH changes. Thus, at low pH values, the release of the encapsulated drug is almost complete after 31 h, whereas at neutral pH, the release is hindered, and only 3% of the initially encapsulated drug is released after 48 h.

Pickering emulsions, i.e., emulsions stabilized by colloidal particles, have also been used as templates for LbL deposition. These emulsions offer as a main advantage their higher stability in relation to conventional ones due to the steric hindrance or electrostatic repulsions associated with the trapping of particles at the fluid/fluid interface, which prevents or at least minimizes the coalescence phenomena. Furthermore, particle adsorption at fluid/fluid interfaces is in most of the cases an irreversible process (trapping energy is much higher than thermal energy), which makes Pickering emulsions extremely stable emulsions [303,304,305,306,307,308,309,310,311,312,313]. The potential of Pickering emulsions as templates for LbL multilayers was proven by Li and Stöver [206]. They prepared xylene in water emulsions stabilized by PSS-decorated nanoparticles, which were used as a template for the fabrication of two type of multilayers: (i) (PDADMAC-PSS)_n_, and (ii) (PDADMAC-silica nanoparticles)_n_. The former multilayers present porous walls and a very significant breakage. On the other side, the hybrid multilayers formed by PDADMAC and silica nanoparticles showed a much higher stability due to their reduced porosity. This allows fabricating platforms for encapsulating a broad range of hydrophobic compounds. Liu et al. [208] also used Pickering emulsions of laponite decorated with polyethyleneimine (PEI) as template for the deposition of (ALG-CHI)_n_ multilayers. They found that the use of this approach allows encapsulating up to five different hydrophobic substances: xylene, chloroform, sunflower oil, dodecane, and liquid paraffin. The modification of the oil phase allows tuning and was found to be a very important parameter for controlling the dimensions of the obtained capsules. Furthermore, Liu et al. [208] demonstrated that polyelectrolyte-decorated Pickering emulsions may be used as templates for preparing hollow capsules. This was possible by dissolving the oil phase in 2-propanol and centrifugation of the dispersion.

Palamarchuk et al. [314] fabricated capsules based on coating Pickering emulsion droplets stabilized by silica nanoparticles with alternate layers of CHI and ALG. They found the existence of two mechanisms for improving the mechanical stability of the obtained capsules: (i) increasing the number of deposited bilayers, and (ii) gelation of the ALG layer by the addition of Ca^2+^ ions.

## 6. LbL Multilayers on Cells

**Figure 13:**
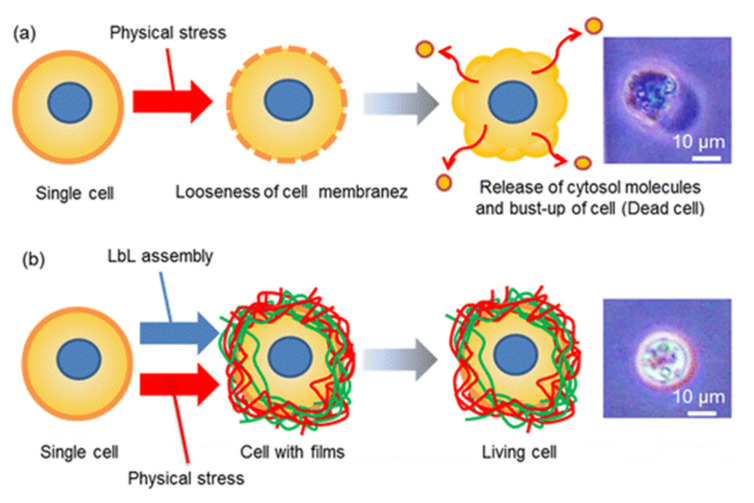
Schematic representation of the positive effect of LbL films on the stability of cells against physical stresses: (**a**) effect of physical stresses on a nude cell and (**b**) effect of physical stresses on a LbL coated cell. Reprinted with permission from reference [315]. Copyright (2013), with permission from American Chemical Society.

The coating of the external surface of cells with LbL multilayers has been extensively explored in tissue engineering for ensuring that cells can face the different stresses from physical, chemical, and environmental origin appearing during their manipulation [315,316]. Figure 13 displays a sketch of the potential improvement of the stability of the cell upon coating with LbL multilayers.

**Figure 14:**
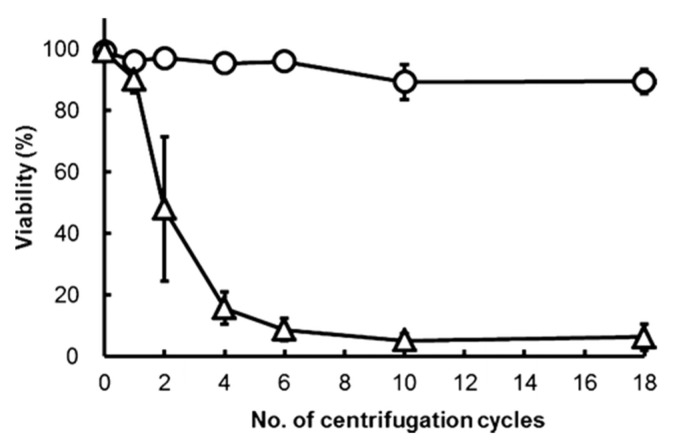
Cell viability after centrifugation for human hepatocyte carcinoma cells with (○) and without (Δ) fibronectin–gelatin containing nine layers. Reprinted with permission from reference [315]. Copyright (2013), with permission from American Chemical Society.

Matsuzawa et al. [315] explored the effect of the coating of human hepatocyte carcinoma cells with multilayers of fibronectin and gelatin, and they found that the viability of the coated cells was about 86% of the initial population after 18 cycles of centrifugation. On the other side, the viability of uncoated cells was reduced to one-half of the initial population after only two centrifugation cycles, and it dropped down to 6% after 18 cycles. Thus, the cell viability was found to be 14-fold increased as result of the protection provided by the deposition of a multilayer containing nine layers. Figure 14 displays the cell viability upon centrifugation for coated and uncoated cells. Amano et al. [317] used the LbL method for coating human induced pluripotent stem cells (iPSCs), which were used for producing human cardiomyocyte (CM) tissues.

Fukuda et al. [316] used the LbL approach for fabricating pancreatic β-cell spheroids taking as a template insulin-secreting MIN6 cells. This was possible by coating the initial cells with multilayers formed by the assembly of fibronectin and gelatin layers. The coated spheroids were found to enhance the glucose uptaking into the pancreatic β-cells, which leads to a higher insulin secretion as a response to glucose stimulation present in relation to uncoated spheroids, with this secretion being more important for spheroids capped with positively charged layers. Furthermore, it was found that the transplantation of the coated spheroids into diabetic mice is an effective tool for disease treatment. This is possible because LbL-coated spheroids are able to promote the expression of Cx36, which is involved in the biosynthesis and release of insulin; hence, its expression results in better insulin secretory responsiveness of pancreatic β-cells.

The application of the LbL technique to cells can provide protection to the cells against physical stresses and a suitable microenvironment capable of regulating the cellular architecture and exerting its function. Furthermore, the coatings of cells using LbL films may be a clinically feasible technique in terms of its simplicity and biological safety.

## 7. Perspectives

The above discussion has evidenced that the combination of the LbL approach with colloidal soft materials opens new possibilities for the transport and controlled release of active molecules, remaining as a very promising tool for enhancing both the versatility and functionality of different formulations, especially with therapeutic interest. However, the true implementation of the LbL systems studied in this review remains far from real. This is firstly a consequence of the difficulties associated with making scalable the production of LbL systems, which is critical to reproducibility and streamlining preparation. Therefore, it is urgent to advance the design of approaches enabling the manufacture of functional platforms and devices. Thus, it will possible to exploit LbL capsules formed by coating soft colloidal nanosurfaces for opening new therapeutic avenues on the delivery of different drugs. This will be possible by combining layers of different nature, taking advantage of the versatility and modularity of the LbL method. Thus, an ideal multi-functional system can include an inner coating without any interaction with the encapsulated molecules, ensuring the protecting role and avoiding any modification on the functionality of the encapsulated molecules and intermediate region, facilitating a controlled and sustained release of the encapsulated molecules, which may be triggered by an external stimuli. These systems may be capped by an external region, which should be modified in such a way that allows the transport of the capsule through the bloodstream without being recognized by the immune system as a risk for health. Furthermore, the controlled targeting, including both the control of the transport process to the target cell, organ, or tissue, and the delivery to the specific target, should be also considered in the design of new LbL platforms for the delivery of therapeutics. In particular, the design of strategies for a local delivery and the control of the interactions with the local environment should be very important challenges to overcome in the future developments of the LbL technology. This requires work on the modulation of the biocompatibility and biodegradability of the obtained platform, and the understanding of the interactions of LbL systems with biological systems to shed light on how the biological response is influenced by the physicochemical parameters of the capsules (elasticity, shape, and size). Last but not least, before translating the designed platforms to routine clinical practice, a full characterization of their safety profiles is necessary.

In summary, the development of LbL platforms based on soft colloidal nanosurfaces is still in its first stage, and many technical challenges remain to make them common tools for clinical applications. However, the strong advance of the LbL method in recent years does not make it difficult to understand that the design of LbL platforms will continue to enable the design of sophisticated materials.

## 8. Concluding Remarks

The LbL method was developed over a long time as a methodology for the assembly of multilayered systems using flat macroscopic substrates or hard colloidal particles as templates. This has allowed developing its potential on diverse technological areas, including the fabrication of multilayered reactors, conducting electrodes, and stimuli-responsive drug release devices. However, the conformation of most of the pioneering supramolecular systems fabricated by the LbL method has limited their efficiency, especially when their applications on the fabrication of carriers and nanoreactors is concerned. This results from the absence on such LbL devices of specific sites to include cargoes or reactants, or when it is possible to fabricate controlled environments for including cargoes (hollow capsules), the existence of residues of the fabrication process can appear (“dirty capsules”). This has created an interest in applying the versatility and modularity of the LbL method to soft colloidal nanosurfaces (e.g., liposomes and vesicles, emulsion droplets, or micelles). The main advantage of these substrates is that they contain one or several pre-formed environments enabling the inclusion of different cargoes, many times of different philicity, e.g., liposomes. Thus, these multilayered structures based on soft colloidal nanosurfaces open exciting avenues for designing engineered multifunctional nanomaterials with application potential in a broad range of technological and industrial fields, including biomedical, cosmetics, food science, or synthetic chemistry, with LbL-decorated soft colloidal nanosurfaces offering a powerful platform for cargo encapsulation, triggered release, and multi compartmentalized hierarchical devices.

It is true that some significant developments in systems based on LbL-decorated colloidal nanosurfaces have been achieved. However, the design of this type of supramolecular system is still in its infancy, and more work is required to understand the main parameter governing the physicochemical properties of the obtained supramolecular systems and their interactions with biological systems (safety profiles). The understanding of such aspects will allow a rational design of a broad range of nanocarriers and nanoreactors that can be introduced into routine practices. However, their true application remains far away, and more research should be performed on the design, optimization, and engineering of formulations based on LbL multilayer-decorated soft colloidal nanosurfaces. Furthermore, the improvement of the modes for remote and/or controlled release and the biodegradability together with the control of the size and aggregation of the supramolecular devices should be important challenges toward the commercialization of these promising systems.

## Data Availability

There are no data associated with this publication.
